# Heat Stress Alters the Intestinal Microbiota and Metabolomic Profiles in Mice

**DOI:** 10.3389/fmicb.2021.706772

**Published:** 2021-08-20

**Authors:** Chaoyue Wen, Siyu Li, Jiaojiao Wang, Yimin Zhu, Xin Zong, Yizhen Wang, Mingliang Jin

**Affiliations:** ^1^Institute of Feed Science, College of Animal Sciences, Zhejiang University, Hangzhou, China; ^2^Key Laboratory of Animal Feed and Nutrition of Zhejiang Province, College of Animal Sciences, Zhejiang University, Hangzhou, China; ^3^Key Laboratory of Molecular Animal Nutrition, Ministry of Education, College of Animal Sciences, Zhejiang University, Hangzhou, China; ^4^Key Laboratory of Animal Nutrition and Feed Science in Eastern China, Ministry of Agriculture, College of Animal Sciences, Zhejiang University, Hangzhou, China; ^5^School of Life Sciences, Northwestern Polytechnical University, Xi’an, China

**Keywords:** heat stress, gut microbiota, metabolomics, fatty acids, SFB

## Abstract

**Background:**

Heat stress has negative effects on the intestinal health of humans and animals. However, the impact of heat stress on intestinal microbial and metabolic changes remains elusive. Here, we investigated the cecal microbial and metabolic profiles in mice in response to heat stress.

**Methods:**

The mouse heat stress model was constructed by simulating a high-temperature environment. Twenty mice were randomly assigned to two groups, the control group (CON, 25°C) and the heat treatment group (HS, 40°C from 13:00 to 15:00 every day for 7 days). Serum and cecal contents were collected from the mice for serum biochemical analysis, 16S rRNA high-throughput sequencing, and non-targeted metabolomics.

**Results:**

Both core body temperature and water intake were significantly increased in the HS group. Serum biochemical indicators were also affected, including significantly increased triglyceride and decreased low-density lipoprotein in the heat stress group. The composition and structure of intestinal microbiota were remarkably altered in the HS group. At the species level, the relative abundance of *Candidatus Arthromitus* sp. *SFB-mouse-Japan* and *Lactobacillus murinus* significantly reduced, while that of *Lachnospiraceae bacterium 3-1* obviously increased after HS. Metabolomic analysis of the cecal contents clearly distinguished metabolite changes between the groups. The significantly different metabolites identified were mainly involved in the fatty acid synthesis, purine metabolism, fatty acid metabolism, cyanoamino acid metabolism, glyceride metabolism, and plasmalogen synthesis.

**Conclusion:**

In summary, high temperature disrupted the homeostatic balance of the intestinal microbiota in mice and also induced significant alterations in intestinal metabolites. This study provides a basis for treating intestinal disorders caused by elevated temperature in humans and animals and can further formulate nutritional countermeasures to reduce heat stress-induced damage.

## Introduction

High ambient temperature is the main factor threatening animal production in tropical and subtropical regions (Mueller et al., 2014; Slimen et al., 2015). Numerous studies have indicated that high temperatures impact not only the growth performance but also immune and intestinal mucosal barrier function in livestock (St-Pierre et al., 2003; Lrar and Rostagno, 2013; Faiz-ul et al., 2019) resulting in increased morbidity, mortality, and economic loss.

The stress response can trigger the organism’s defense system, mitigating the damage caused by the stressor and maintaining physiological balance (Wen et al., 2019, 2020a). In the case of excessive stress or long-term stress on the organism, the stress response will gradually weaken and finally present as a pathological state (Wen et al., 2020b). Heat stress can seriously damage the intestinal tract, significantly increasing intestinal permeability (Cui and Gu, 2015; Zhang et al., 2017). Heat stress can also affect the immune function, potentially leading to intestinal mucosal damage (Gu et al., 2012; Tao et al., 2012). Through the study of the intestinal contents of heat-stressed broilers, it was found that the viable counts of *Lactobacillus* and *Bifidobacterium* were significantly reduced, resulting in an imbalance of intestinal microecology (Song et al., 2013, 2014; Al-Fataftah and Abdelqader, 2014).

The host metabolism is altered in response to environmental changes, specifically in terms of metabolic adaptations (Virtue et al., 2019). For example, a reduction in food intake can result in shortening of the jejunum (Lemme and Mitchell, 2008; Payne, 2019). The level of serum triglyceride (TG) was found to be significantly lower under chronic heat stress (He et al., 2019b). Water intake is an efficient way to alleviate heat stress, resulting in a lower rectal temperature and respiration rate (Marai et al., 2001). In broilers, both the total water intake and water intake per access were significantly increased at a high-temperature house (Bruno et al., 2011). Postabsorptive carbohydrate and lipid metabolism are also markedly altered (Baumgard and Rhoad, 2012). Together, these results suggested that heat stress exerts a negative effect on an organism’s metabolism.

The microbiota appears to play an important role in the stress response (Sekirov et al., 2010; Zong et al., 2020) and the microbiota composition is related to heat tolerance (Ziegler et al., 2017). These intestinal microorganisms can assist in the maintenance of the intestinal barrier, thus effectively ensuring the host’s health (Guarner and Malagelada, 2003). Disruption of the intestinal temperature may allow pathogen invasion and the consequent development of disease (Harvell et al., 2002). Although heat treatment has no great effect on the alpha diversity of the microbiome, alterations at the phylum and genus levels were observed (Zhong et al., 2019). Segmented filamentous bacteria (SFB) are host-specific gut symbionts that induce a multifaceted immune response, leading to host protection from gut pathogens (Pamp et al., 2012). It has been found that SFB can prevent the colonization of enteropathogenic *Escherichia coli* O103 (Heczko et al., 2000). Moreover, SFB is involved in lipid metabolism (Nguyen et al., 2007). Homeostatic disturbance of the gut microbiota may cause abnormal growth of microorganisms and inadequate absorption of host nutrients that be captured by microorganisms.

Heat stress has a deleterious effect on human and animal welfare and causes economic losses in livestock production. Therefore, in the present study, a mouse model was used to investigate the impact of heat stress on the diversity and metabolism of intestinal microbiota by next-generation sequencing and GC-TOF/MS. The aim was to explore the effects of heat stress on intestinal microbial diversity, metabolism, physiological and biochemical parameters in mice. Correlation analysis was used to determine the relationship between regulatory processes induced by heat stress and the intestinal microbiome community, thus providing a theoretical and experimental basis for our understanding of the effects of high temperature on humans and animals.

## Materials and Methods

### Animal Experiments

All animal experiments in the present study were approved by the Institutional Animal Care and Use Committee of Northwestern Polytechnical University, China, and performed following the institutional ethical guideline of experimental animals. Adult female ICR mice (30.2 ± 2.5 g) aged 7 weeks were purchased from the Animal Experimental Center of Xi’an Jiaotong University, China. The heat stress model was established according to previous study (Minho et al., 2017; Chen et al., 2020). The core temperature of mice is 37 ± 1°C. Under conditions when the increasing temperature is beyond the upper critical temperature of the range, the animals begin to suffer heat stress (Rojas-Downing et al., 2017). The hottest time of the day is between 1 and 3 PM. Taking these together, we chosen 40°C lasted 2 h as the heat stress condition. All animals had free access to food and drinking water and were housed in plastic cages in a controlled environment (temperature, 25°C; relative humidity, 60%; lighting cycle, 12 h/d). After 10 days of acclimatization under normal conditions, a total of 20 mice were randomly assigned to two groups (*n* = 10), including the control group (CON) and the heat stress group (HS). Starting from the 11th day, the temperature of the HS group was raised to 40°C from 13:00 to 15:00 during feeding every day and returned to 25°C for the remainder of the day. The experiment lasted for 7 days. At the end of the experiment, the animals were anesthetized and blood was withdrawn by orbital bleeding. Serum samples were separated after centrifugation at 4,000 × *g* for 15 min at 4°C. The cecum contents were also collected for 16S rRNA sequencing and GC-TOF/MS analysis.

### Body Weight, Water Intake, and Core Body Temperature

After the HS treatment, the body weights were measured every 2 days. The daily water intake of the mice was also determined.

### Serum Biochemical Parameters

The serum biochemical parameters included total cholesterol, TG, high-density lipoprotein, and low-density lipoprotein (LDL) were investigated using an automatic biochemical analyzer (Shenzhen Redbang Electronics Co., Ltd., China).

### DNA Extraction, Library Construction, and Sequencing

Total DNA from the cecal contents was extracted using the E.Z.N.A.^®^Genomic DNA Isolation Kit (Omega Bio-Tek, United States) according to the manufacturer’s instructions. To investigate the bacterial community structure, we used next-generation 16S rRNA sequencing to analyze the composition of the cecal microbiota. The V3-V4 hypervariable region of the bacterial 16S rRNA gene in each sample was amplified using the broadly conserved primers, 27F (5′-AGAGTTTGATCCTGGCTCAG-3′) and 533R (5′-TTACCGCGGCTGCTGGCAC-3′), and then sequenced using an Illumina MiSeq PE250 (Illumina, San Diego, CA, United States). The assembled MiSeq sequences were submitted to the NCBI’S Sequence Read Archive (SRA BioProject No. PRJNA730381) for open access. The resulting raw sequences were filtered and assembled according to previous research (Jin et al., 2019; Zhou et al., 2019) and using the QIIME (v1.9.1) and FLASH (v1.2.11) software packages. The filtered sequences were compared with SILVA (v132) small subunit ribosomal RNA database, and the similarity more than 80% of the species information was screened out. In the taxonomic analysis of each OTU, sequences with 97% similarity were selected first, and the consistency of these sequences was analyzed. Finally, the species information of each OTU was taken as the species information of its nearest ancestor. The analysis and production of rarefaction curves were performed by Mothur (v1.30.2) and R software, respectively. To investigate bacterial richness and diversity, Mothur was also used to analyze the alpha diversity, including the Chao, Ace, Shannon, and Simpson indices. The OTU coverage curves were expressed using the “vegan” R package.

### Sample Preparation and GC-TOF/MS Analysis

Cecal samples (100 μL) were slowly thawed at 4°C. 200 μL acetonitrile was added, followed by sonication for 10 min, and centrifugation at 10,000 × *g* for 10 min at 4°C. The supernatant was removed and vacuum-dried at 40°C. For mass spectrometry, 50 μL 15 mg/mL methoxyamine pyridine solution was reconstituted, vortexed, and incubated at 70°C for 1 h. 50 μL silanization reagent (MSTFA: TMCS = 100:1) was added to the centrifuge tube for derivatization, mixed well, allowed to stand for 1 h, and then added to a concentration of 0.1 mg/mL n-heptane containing 150 μL docosane, before thorough mixing and centrifugation at 10,000 × *g* for 10 min at 4°C. The supernatant was retained and transferred to a sample bottle for GC-TOF/MS analysis (Agilent 7890A gas chromatograph equipped with an Agilent DB-5MS capillary column (30 m × 250 μm × 0.25 μm, J&W Scientific, Folsom, CA, United States).

The derivatized sample (1.0 μL) was injected by the splitless mode. The helium carrier gas flow rate was 1 mL/min. The oven temperature ramp program was set as follows: initial holding at 50°C for 1 min, increasing to 240°C at 10°C/min, and finally holding for 2 min. The temperatures of the front inlet, transfer line, and ion source were set at 280, 270, and 220°C, respectively. The ionization voltage was set to − 70 eV, the quality control ranged from 50 to 500 *m/z*, the scan rate was 20 spectra/s, and the solvent delay time was 6 min.

### GC-TOF/MS Data Processing and Differential Metabolites Identification

After the raw data was collected, the LECO’s ChromaTOF software was used for peak alignment, retention time correction, deconvolution analysis, peak identification, and area extraction. Principle component analysis (PCA) and orthogonal partial least squares method-discriminant analysis (OPLS-DA) were performed using SIMCA software. After the analyzed data was matched with the KEGG data ID, path enrichment and network construction were performed (Haug et al., 2019). Analysis of differential metabolites and metabolic pathways were performed based on MetaboAnalyst 4.0.^1^ The RAW data of GC–TOF/MS has been submitted to Metabolights NO. MTBLS2874.^2^

### Data Analysis

All statistical analyses were performed using SAS 8.2 software (SAS Institute, Inc.). The data relating to the microbiota community were analyzed on the free online platform of Majorbio Cloud Platform.^3^ Metagenomes were predicted from the copy number-normalized 16S rRNA data according to the previous report (Zhou et al., 2018). The molecular functions were categorized into KEGG pathways on the web-based Galaxy according to the instructions described by developers.^4^ ^5^ Correlation analysis was computed with spearman test in R using corrplot package (Wei and Simko, 2013). The codes used in the analysis could be found on the websites.^6^ Details can be found in the legends of the corresponding figures and tables. The difference between CON and HS was compared using an unpaired *t*-test. *P*-values < 0.05 indicated statistical significance.

## Results

### Body Weight, Water Intake, and Core Body Temperature

**FIGURE 1:**
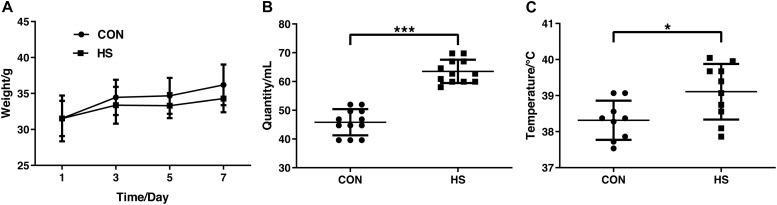
Changes in body weight **(A)** water intake **(B)** and core body temperature **(C)** on day 7. CON, control group; HS, heat stress group. *P < 0.05, ***P < 0.001.

In the heat stress mouse model, no significant difference was found in body weight between the groups (Figure 1A). As expected, both the relative intake of water (Figure 1B) on the 7th day and the body temperature significantly increased in the HS group (Figure 1C) (*n* = 10 per treatment). This indicated the successful establishment of the heat stress model.

### Serum Biochemical Indices

**FIGURE 2:**
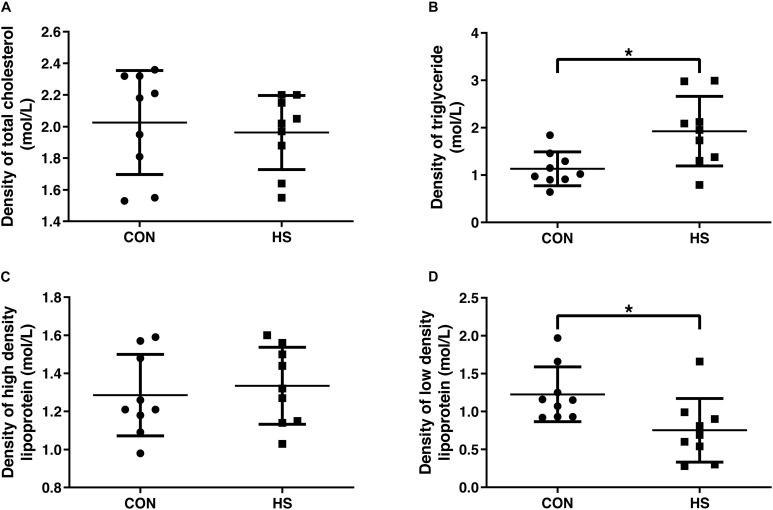
Changes of blood lipid indicators. Density of total cholesterol **(A)**, triglyceride **(B)**, high density lipoprotein **(C)** and low density lipoprotein **(D)** in serum. CON, control group; HS, heat stress group. *P < 0.05.

We further analyzed serum concentrations of lipids. The fasting serum lipids values are presented in Figure 2. There were no significant changes in the levels of total cholesterol and high-density lipoprotein (Figures 2A, C). Compared with the CON group, the level of serum TG increased significantly, and LDL decreased remarkably in the HS group (Figures 2B, D) (*n* = 10 per treatment).

### Cecal Microbial Community

Across all 20 samples, 1451173 high-quality sequences were identified, with an average length of 411 bp. No remarkable differences were found in the richness estimators (Ace and Chao), diversity indices (Shannon and Simpson), and observed OTUs (Supplementary Table 1). The normalized microbiome data has been added in the supplementary material (Supplementary Table 2).

**FIGURE 3:**
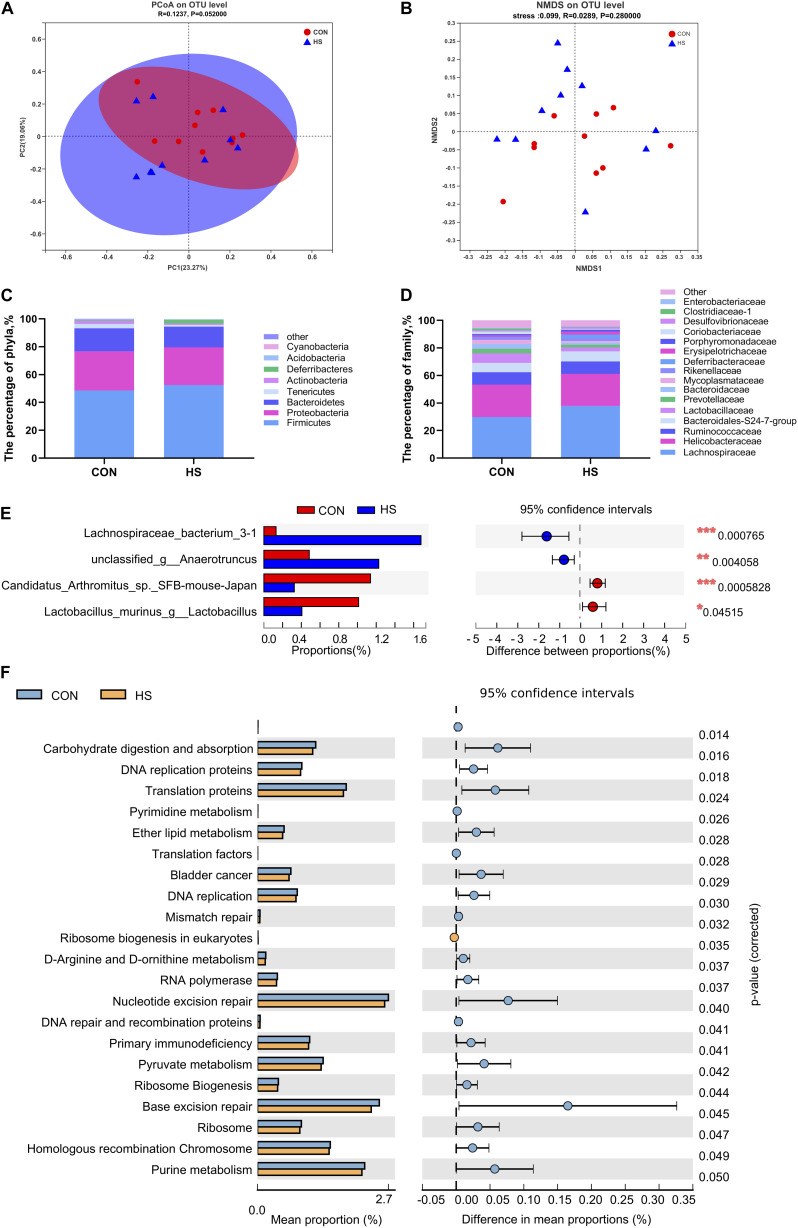
Effects of heat stress on mouse gut microbiota. **(A)** Analysis of the PCoA plots based on a Bray-Curtis distance metric. **(B)** NMDS ordination plots of cecal bacterial communications in the HS and CON group based on the Bray-Curtis distance metric. **(C)** Differential microorganisms at the phylum level. **(D)** Differential microorganisms at the family level. **(E)** *T*-test bar plot of significantly different species between the groups (relative abundance > 1%). **(F)** *T*-test bar plot of significantly differed pathways enriched at KEGG level 3. CON, control group; HS, heat stress group; PCoA, principal coordinates analysis; NMDS, Non-metric multidimensional scaling.

We further investigated the shifts in bacterial taxa that were responsible for heat stress adaptation. Principal coordinates analysis (PCoA) based on weighted_unifrac revealed distinct clustering of microbiota composition for the two groups (Figure 3A). Analysis of the similarities in the Bray-Curtis distance indicated that the heat-stressed and control mice tended to be different (*P* = 0.052) with an *R*-value of 0.1237, suggesting that the microbiota of the two groups were different. A non-metric multidimensional scaling (NMDS) ordination plot based on the Bray-Curtis distance metric showed that the cecal bacterial communities in the samples could be differentiated by heat treatment (Figure 3B).

The overall microbial composition of the two groups differed at the phylum, family, genus, and species levels. The five largest phyla represented in each group were Firmicutes, Proteobacteria, Bacteroidetes, Tenericutes, and Deferribacteres. The thermoneutral mice had a higher relative abundance of Proteobacteria (28.1%), Bacteroidetes (16.4%), and Tenericutes (3.1%), but a lower relative abundance of Firmicutes (48.7%) and Deferribacteres (0.5%) (Figure 3C). Heat-treated mice contained largely bacteria of the phyla Bacteroidetes 14.9%, Firmicutes 52.5%, Proteobacteria 26.7%, Tenericutes 1.3%, and Actinobacteria 1.0% (Figure 3C). No statistical differences were observed in the relative abundance of the five largest phyla. However, heat treatment tended to decrease the proportion of Tenericutes and Actinobacteria (*P* = 0.071 and 0.098, respectively) (Figure 3C). These results suggested that the gut microbiota composition of mice remained relatively stable under heat stress. At the family level, *Clostridiaceae_1* was significantly enriched in thermoneutral conditions (Figure 3D and Supplementary Figure 1A). The relative abundance at the levels of class and order were presented in supplementary materials (Supplementary Figures 1D–F). At the genus level, *Lachnospiraceae bacterium 3-1* and *unclassified g-Anaerotruncus* were significantly increased, while *Candidatus Arthromitus* sp. *SFB-mouse-Japan*, *Lachnoclostridium*, and *Lactobacillus murinus* were significantly decreased in the heat-treated mice (Figure 3E and Supplementary Figure 1B). At the OTU level, OTU1468, and OUT1733 were significantly increased in the HS group, while OTU1364 and OTU778 were significantly decreased in the CON group (Supplementary Figure 1C).

### Predicted Molecular Functions of Cecal Microbiota

We found that multiple KEGG (level 3) categories were disturbed in the heat-treated group. The KEGG at level 2 category results were consistent with the findings of KEGG at level 3 (Figure 3F). Specifically, the enriched pathways were membrane transport, lipid metabolism, infectious disease: bacterial, infectious disease: parasitic, immune disease, excretory system, cellular community-prokaryotes, cell motility, glycan biosynthesis, and metabolism, and signaling molecules and interaction (Supplementary Figure 2). Moreover, carbohydrate digestion and absorption, DNA replication proteins, translation proteins, and pyrimidine metabolism were significantly upregulated in the CON group (Figure 3F).

### Variations in Cecal Metabolite Profiles

**FIGURE 4:**
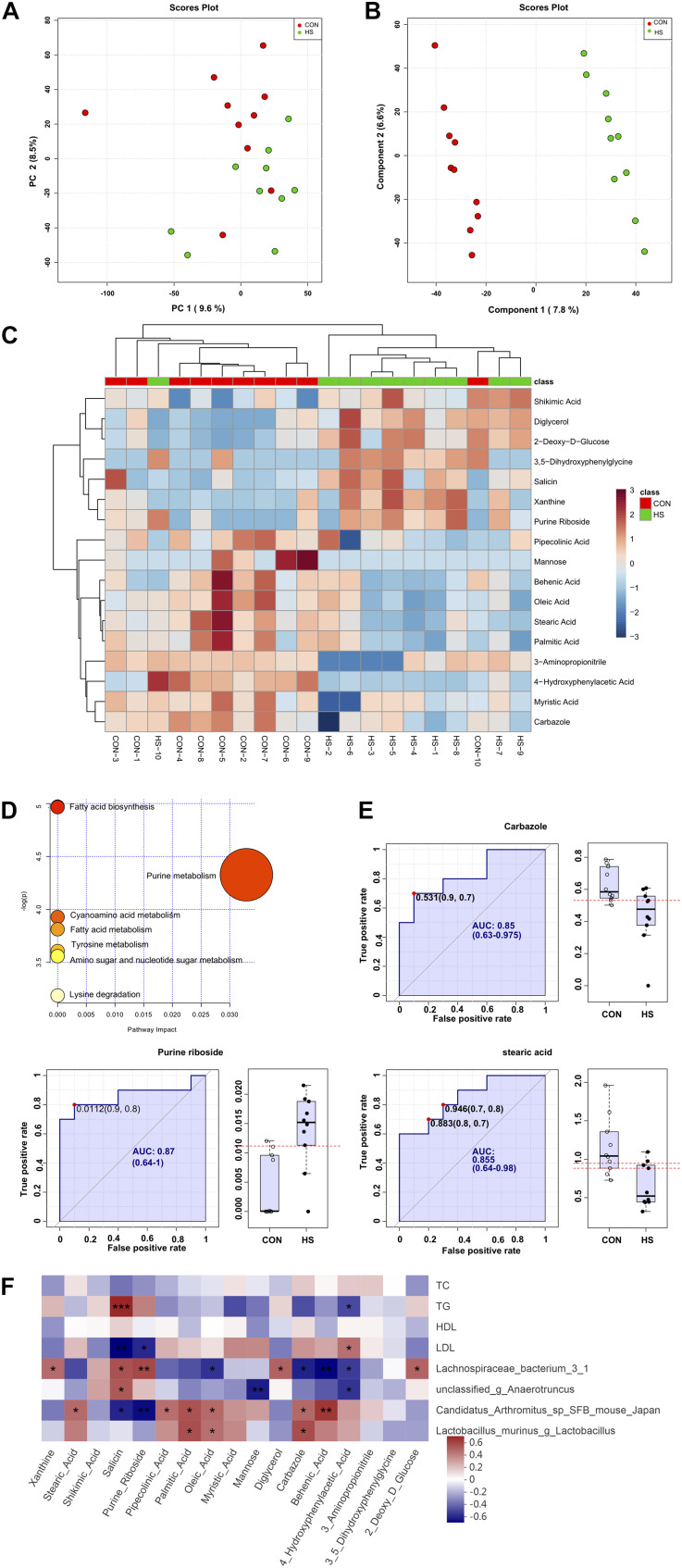
Changes in cecal metabolite profile of mice shaped by heat stress. **(A)** PCA score plot. **(B)** OPLS-DA score plot. **(C)** heatmap tree show metabolites significantly different between groups and their phylogenic relationships. **(D)** Metabolic pathway enrichment analysis. **(E)** Biomarker analysis. **(F)** Correlations between serum indicators and gut microbiota, and cecum metabolic indicators mediated by heat stress. CON, control group; HS, heat stress group; OPLS-DA, Orthogonal partial least squares method-discriminant analysis. *P < 0.05, **P < 0.01, ***P < 0.001.

To explore the effects of heat treatment on cecal metabolites in mice, GC-TOF/MS was applied to investigate the intestinal metabolite profiles. A total of 532 effective peaks were obtained, of which 235 compounds were relatively quantified, 120 were labeled “analyte,” 173 were labeled “unknown” as compared against the LECO-Fiehn Rtx5 database. The differences of metabolomics profiles between CON and HS groups by the multivariate analysis are shown in Figures 4A, B. Principal component analysis (PCA) of the metabolites showed no clear distinction between the groups (Figure 4A). To further verify the differences between the groups, we did an OPLS-DA which clearly distinguished the metabolites (Figure 4B). It indicated that the GC-TOF/MS-based metabolomics and PLS-DA model was suitable to be applied in identifying the differences between the two groups. Furthermore, the heatmap showed significant changes in the intestinal metabolites between the two groups (Figure 4C). Compared with the CON, the heat stress group showed significant up-and down-regulation of 7 metabolites and 10 metabolites, respectively (Supplementary Table 3). In these metabolites, xanthine, shikimic acid, salicin, purine riboside, diglycerol, 3,5-dihydroxyphenylglycine, and 2-deoxy-D-glucose were enriched in the HS group. Conversely, there was a increase of metabolites such as stearic acid, pipecolinic acid, palmitic acid, oleic acid, myristic acid, mannose, carbazole, behenic acid, 4-hydroxyphenylacetic acid, and 3-aminopropionitrile in the CON group (Supplementary Table 3). The normalized metabolomics data is provided in spreadsheets on the supplementary material (Supplementary Table 4).

### Differential Metabolic Pathway Analysis

KEGG analysis of the 17 significantly different metabolites showed enrichment of the fatty acid biosynthesis, purine metabolism, fatty acid metabolism, cyanoamino acid metabolism, tyrosine metabolism, amino sugar and nucleotide sugar metabolism, and lysine degradation pathways (Figure 4D). Further analysis of the metabolic pathways by bubble diagram and metabolic pathway enrichment revealed that heat treatment significantly inhibited the fatty acid synthesis pathway.

### Analysis and Verification of Biomarkers

Based on variable importance for projection (VIP) > 1.0 in the OPLS-DA and *P*-value < 0.05 between the two groups, carbazole, purine nucleoside, and stearic acid were selected as biomarkers in the intestine that responded to heat stress (Figure 4E). The AUCs of carbazole (AUC value = 0.85), purine nucleoside (AUC value = 0.87), and stearic acid (AUC value = 0.855), as well as the levels of these three compounds in the intestine, are shown in Figure 4E. Besides, cross-validation prediction of the samples with three metabolites indicated that CON and HS showed a significant separation trend (Supplementary Figure 3). The results of comprehensive prediction with these three biomarkers showed that the average AUC was 0.973, which was extremely close to 1 (Supplementary Figure 3), and that the two groups of samples were clear separated and discriminated.

### Correlation Analysis Between Serum Index, Significantly Different Microbiota and Metabolites

To further study the correlation between gut microbiota, metabolites, and serum biochemical markers, Spearman analyses were performed (Figure 4F). It was observed that TG showed a significant negative correlation with 4-hydroxyphenylacetic acid (ρ = −0.45, *P* = 0.049) and a positive correlation with the salicin level (ρ = 0.68, *P* < 0.001). However, the correlation between LDL and 4-hydroxyphenylacetic acid (ρ = 0.46, *P* = 0.043) and salicin (ρ = −0.63, *P* = 0.003) level was exactly the opposite of TG.

There is a close relationship between gut microbiota and metabolites. For instance, *Candidatus Arthromitus* sp. *SFB-mouse-Japan* showed a negative correlation with salicin (ρ = −0.55, *P* = 0.013) and purine riboside (ρ = −0.60, *P* = 0.005) and a positive correlation with behenic acid (ρ = 0.63, *P* = 0.003). *Lactobacillus murinus* was positively correlated with carbazole (ρ = 0.56, *P* = 0.010) and palmitic acid (ρ = 0.55, *P* = 0.011). Moreover, *Lachnospiraceae bacterium 3-1* was positively associated with salicin (ρ = 0.54, *P* = 0.013), purine riboside (ρ = 0.58, *P* = 0.008), and diglycerol (ρ = 0.50, *P* = 0.026, but negatively associated with behenic acid (ρ = −0.62, *P* = 0.004), oleic acid (ρ = −0.51, *P* = 0.022), and carbazole (ρ = −0.56, *P* = 0.011). *Unclassified-g-Anaerotruncus* was positively associated with salicin (ρ = 0.52, *P* = 0.018) but negatively correlated with both mannose (ρ = −0.57, *P* = 0.009) and 4-hydroxyphenylacetic acid (ρ = −0.50, *P* = 0.024).

## Discussion

Temperature is a crucial environmental signal that controls the growth and development of bacteria. Continuous high temperature may cause functional disorders, including intestinal dysbiosis (Bouchama and Knochel, 2002; Kovats and Hajat, 2008; He et al., 2019a). The impact of heat stress on microbial composition and metabolites in mice is still limited. A better understanding of the physiological alterations of the microbial community and its metabolites under heat stress could help to develop targeted approaches to alleviate heat stress.

Heat stress induces weight loss was reported in ducks (He et al., 2019b), broiler (Luo et al., 2018). In our study, we also found a body weight loss in the HS group. Animals developed a phenotypic response to heat acclimation which results in decreased feed intake and increased water intake to accommodate increased evaporative heat loss requirements (Robert et al., 2018). The core temperature is significantly increased in the HS group, which indicated that our heat stress model was successfully established.

Blood biochemical indicators can be used to determine metabolic status. It was found that the TG content was significantly increased with the LDL level showing an opposite trend in HS mice. LDL is mainly responsible for transporting cholesterol from the liver to the tissues of the body, and then metabolizing cholesterol by the body (Cirulli and Ginsburg, 2017). When the body is subjected to heat stress, cholesterol metabolism is slowed down, resulting in a decrease in LDL levels. In broilers, heat stress was found to increase the TG concentration (Luo et al., 2018), consistent with our results. In general, there is an alteration in the metabolic response to heat stress characterized by an increase in the use of carbohydrates and a decrease in fat usage (Febbraio, 2001). It follows that the TG content in the blood is significantly increased.

The effect of heat stress on the intestinal microbiota and their microecological structure in mice was studied by 16S rRNA high-throughput sequencing. Analysis of the rarefaction curves and alpha diversity showed that there were no significant differences in the alpha diversity indices, indicating that HS had no significant effect on intestinal microbiota diversity. According to the sequencing, the mouse intestinal microbiome consists mainly of five phyla, the Firmicutes, Proteobacteria, Bacteroidetes, Tenericutes, and Deferribacteres. Among these, the dominant microbial group was Firmicutes, accounting for more than 60% of the microbiome. Besides these, we also detected Verrucomicrobia, Acidobacteria, Actinomycetes, and Cyanobacteria in the mouse intestine, with low proportions of less than 0.2%. The Firmicutes/Bacteroidetes ratio, a parameter to evaluate the imbalance of microbial composition, has been used to indicate obesity in the host (Wen et al., 2008). In our study, the Firmicutes/Bacteroidetes ratio increased by 21.5% in the heat-treated group (Supplementary Figure 1G).

Accumulating evidence has revealed the dysbiosis of intestinal microbiota induced by heat stress in mammals and poultry (Song et al., 2013; Zhang et al., 2017; Zhu et al., 2018; He et al., 2019b). Stress-induced by extreme environments such as simulated weightlessness can also change the composition of the intestinal microbiota, decrease the diversity of intestinal microorganisms (Chen et al., 2016), lead to changes in the homeostasis of colonic epithelial cells and barrier function, and also cause pathological changes of the intestinal mechanical barrier, including intestinal villus damage and down-regulation of tight junction protein expression, thereby changing intestinal permeability (Shi et al., 2017; Jin et al., 2018). The present study indicated that heat stress significantly reduced the abundance of *Candidatus Arthromitus* sp. *SFB-mouse-Japan*, *Lactobacillus murinus* in the gut, and significantly increased the abundance of *Lachnospiraceae bacterium 3-1*. *Candidatus Arthromitus* plays an important role in host immune regulation, as it is in contact with epithelial cells and be transplanted into host epithelial cells, thereby triggering a series of physiological responses related to the host immune system (Pamp et al., 2012; Bolotin et al., 2014). SFB are host-specific intestinal symbionts that comprise a distinct clade within the *Clostridiaceae*, designated *Candidatus Arthromitus* (Pamp et al., 2012). SFB induces a multifaceted immune response, leading to host protection from intestinal pathogens (Pamp et al., 2012). *Candidatus Arthromitus* sp. *SFB-mouse-Japan* was one of the five SFB filaments isolated from a mouse (Pamp et al., 2012). SFB has a relatively high abundance of predicted proteins devoted to cell cycle control and to envelope biogenesis (Pamp et al., 2012). The dominance of *Lactobacillus* in the intestine is associated with protection against pathogens and infections (Reid and Burton, 2002). *Lactobacillus* plays an essential role in food fermentation and is used in probiotic applications (Heeney et al., 2018). The most abundant lactobacilli included *L. murinus*, *L. casei* and *L. ruminus*, and *L. murinus* is considered a gut-autochthonous microorganism. *Lactobacillus* has been reported to be remarkably enriched in the distal gut (Rossi et al., 2016). The depletion of intestinal *Lactobacillus* is frequently associated with the disease. *Oscillibacter*, a beneficial bacterium, is significantly reduced in patients (Fang et al., 2016), piglets with intrauterine growth retardation (Zhang et al., 2019), and obese mice (Gong et al., 2020), indicating that the intestinal microflora constitution was disturbed. *Ruminococcaceae* is significantly higher in the intestinal flora of a high-risk colorectal cancer population than in a low-risk population (Moore and Moore, 1995). It was speculated that a high-temperature environment may have specific effects on patients with colorectal cancer. *Lachnospiraceae* are the main producers of short-chain fatty acids (SCFAs) and have been associated with intestinal diseases (Vacca et al., 2020). They are also increased in obese subjects, which suggested that the metabolic syndrome may be related to a gut microbiota disorder (Zhao et al., 2019). The study of microbial excavation and interaction is useful to reveal the influence of heat stress on the intestinal mucosal barrier and can provide a theoretical basis and experimental ideas for the prevention and repair of body damage caused by heat stress. The gut flora plays a key role in host energy metabolism (Guarner and Malagelada, 2003; Dai et al., 2011; Pérez-Cobas et al., 2013). Indigestible carbohydrates are degraded by fermentation of colonic microflora to produce metabolic end products, such as SCFAs, these metabolites have been shown to affect host physiological activities (Topping and Clifton, 2001; Wong et al., 2006; Wang et al., 2019; Guo et al., 2020). Recent studies have confirmed that SCFAs can inhibit the production of anti-inflammatory factors and inhibit colonic inflammation (Tan et al., 2014; van der Beek et al., 2017; Kurata et al., 2019; Zeng et al., 2019).

To determine the effect of heat stress on the mice’s cecal metabolites, GC-TOF/MS was used to explore the chemical constituents of the intestinal contents in both groups. The results showed that heat treatment produced significant changes in the cecal metabolites with most metabolites significantly reduced compared with the CON, including oleic acid, palmitic acid, stearic acid, mannose, myristic acid, and carbazole. These metabolites are involved in the physiological and biochemical processes of energy metabolism and lipid metabolism (Savage et al., 2007; Loscalzo, 2011; Li et al., 2017). Heat treatment inhibited fatty acid synthesis, shown by combining a bubble diagram and metabolic pathway enrichment. Meanwhile, three metabolites were screened as biomarkers, namely carbazole, purine nucleoside, and stearic acid, and these were used to cross-validate and predict the HS and CON samples. Salicin showed a potential therapeutic agent against LPS induced acute injury (Li et al., 2015). In our study, the salicin was significantly increased in the HS group (Supplementary Table 3). It suggested that the host triggered an adapted response to counter heat stress-induced inflammatory processes.

Intestinal metabolites are agents between the microbiota and energy metabolism (Karl et al., 2018). Previous study have found that heat stress could induce the increase of fatty acids (Cui et al., 2019). The correlation analysis in this study suggested that TG was significant negative correlation with 4-hydroxyphenylacetic acid and positive correlation with the salicin level Interestingly, the correlation between LDL and 4-hydroxyphenylacetic acid and salicin level was exactly the opposite of TG. *Candidatus Arthromitus* sp. *SFB-mouse-Japan* showed a negative correlation with salicin and purine riboside and a positive correlation with behenic acid. These results indicated that microbiota are involved in the regulation of energy metabolism.

In conclusion, this study revealed the important relationship between intestinal microbiota structure and metabolism under heat stress. We need not only to identify changes in the intestinal flora structure but also to understand the correlation between the microflora and disease under heat treatment. Our study screened some metabolites and microbiota in the cecum of heat-stressed mice might have potential beneficial properties. This study provides a theoretical and experimental basis for further research into high-temperature damage in humans and animals.

## Data Availability Statement

The assembled MiSeq sequences were submitted to the NCBI’S Sequence Read Archive (SRA BioProject No. PRJNA730381) for open access. The RAW data of GC–TOF/MS has been submitted to metabolights no. MTBLS2990 (www.ebi.ac.uk/metabolights/MTBLS2990).

## Ethics Statement

The animal study was reviewed and approved by Institutional Animal Care and Use Committee of Northwestern Polytechnical University, China.

## Author Contributions

MJ designed the experiment. YZ conducted the experiment. CW and MJ collected and analyzed the data. MJ, SL, YW, and XZ helped with the discussion. CW, SL, JW, and MJ wrote and revised the manuscript. All authors contributed to the article and approved the submitted version.

## Conflict of Interest

This study received funding from Shanghai Biotree Biomedical Technology CO., LTD. The funder was involved in the collection of data. All authors declare no other competing interests.

## Publisher’s Note

All claims expressed in this article are solely those of the authors and do not necessarily represent those of their affiliated organizations, or those of the publisher, the editors and the reviewers. Any product that may be evaluated in this article, or claim that may be made by its manufacturer, is not guaranteed or endorsed by the publisher.

## Footnotes

1
http://www.metaboanalyst.ca

2
www.ebi.ac.uk/metabolights/MTBLS2990

3
www.majorbio.com

4
http://huttenhower.sph.harvard.edu/galaxy/

5
http://picrust.github.io/picrust

6
https://github.com/zlabx/zlab-qiime2

## Supplementary Material

Supplementary Figure 1 Effect of heat stress on cecal microbiota of mice. **(A)** Welch’s *t*-test bar plot at the family level (relative abundance >1%). **(B)** Welch’s *t*-test bar plot at the genus level (relative abundance >1%). **(C)** Welch’s *t*-test bar plot at the OTU level (relative abundance >1%). **(D)** Relative abundance of microbiome at the class level. **(E)** Relative abundance of microbiome at the order level. **(F)** Relative abundance of microbiome at the genus level. **(G)** The ratio of Firmicutes/Bacteroidetes. CON=control group; HS=heat stress group.

Supplementary Figure 2 The heatmap of KEGG pathway level 2. CON=control group; HS=heat stress group.

Supplementary Figure 3 Biomarker analysis of metabolites based on the random forests algorithm. ROC view **(A)**, probability view **(B)**, and cross validation **(C)** of three selected metabolites, namely carbazole, purine nucleoside and stearic acid. CON=control group; HS=heat stress group.

Supplementary Table 1 The influence of HS on α-diversity of gut microbiota.

Supplementary Table 2 The normalized microbiome data.

Supplementary Table 3 Significantly different metabolites in cecal contents between CON and HS groups.

Supplementary Table 4 The normalized metabolomics data.

## Abbreviations

AUC: area under the curve
LDL: low-density lipoprotein
OPLS-DA: Orthogonal partial least squares method-discriminant analysis
PCoA: principal coordinates analysis
SCFAs: short-chain fatty acids
SFB: segmented filamentous bacteria
TG: triglyceride.
