# Supplementary figures and images for: Heat Stress Alters the Intestinal Microbiota and Metabolomic Profiles in Mice
Source: Front Microbiol. 2021 Aug 20;12:706772. doi: 10.3389/fmicb.2021.706772 (PMC8430895; doi:10.3389/fmicb.2021.706772)

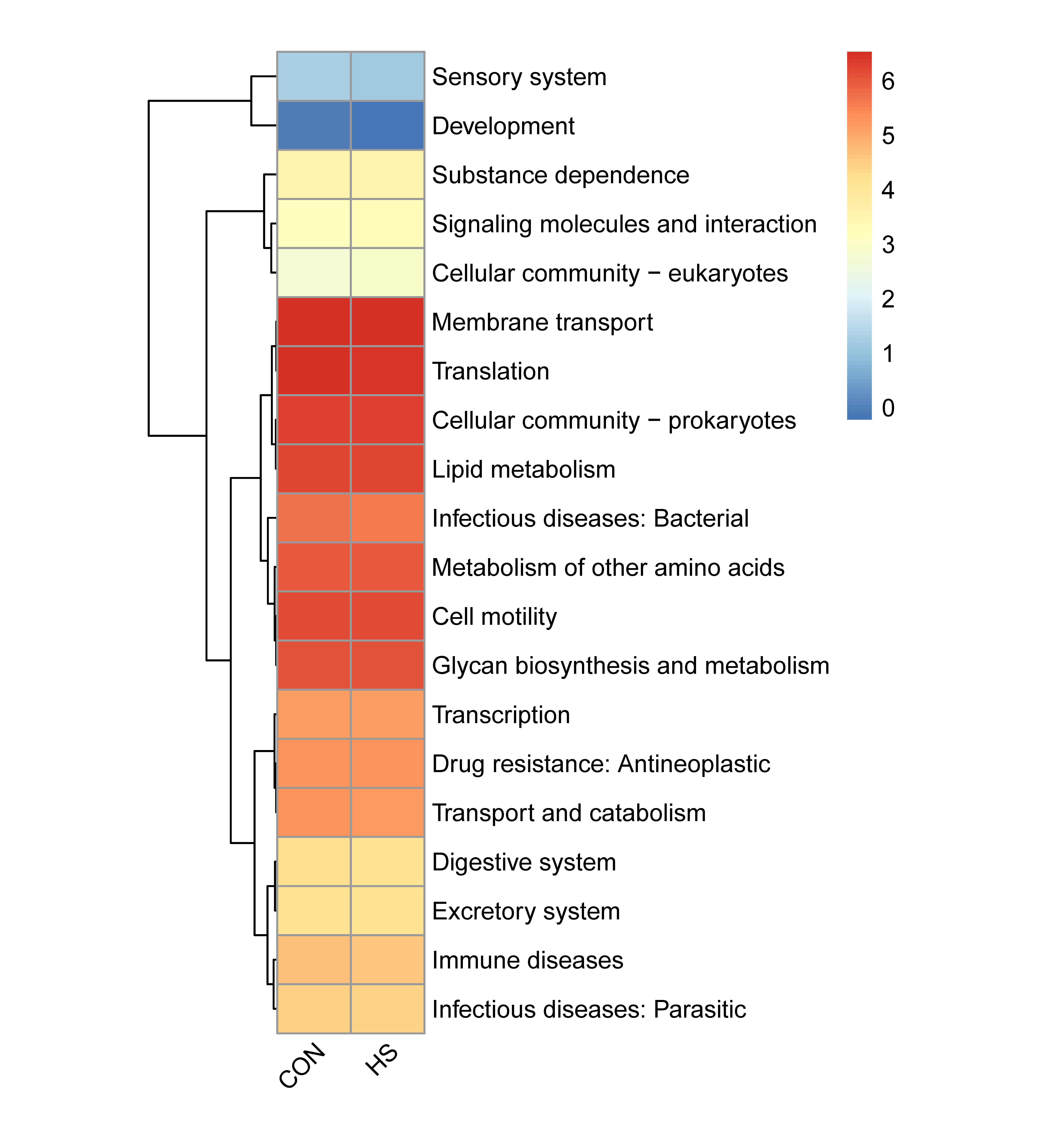

Supplement: Supplementary Figure 2 — The heatmap of KEGG pathway level 2. CON=control group; HS=heat stress group. [file Image_2.tif]
