# Supplementary material for: Heat Stress Alters the Intestinal Microbiota and Metabolomic Profiles in Mice
Source: Front Microbiol. 2021 Aug 20;12:706772. doi: 10.3389/fmicb.2021.706772 (PMC8430895; doi:10.3389/fmicb.2021.706772)
Supplement: Supplementary Table 1 — The influence of HS on α-diversity of gut microbiota. [file Table_1.docx]

Table S1. The influence of HS on α-diversity of gut microbiota

| Items | Groups | | *P*-value |
| --- | --- | --- | --- |
|  | CON | HS |  |
| Ace | 820.20±176.21 | 821.20±120.96 | 0.61 |
| Chao | 807.10±174.83 | 809.30±121.08 | 0.69 |
| Shannon | 5.80±0.76 | 5.85±0.96 | 0.75 |
| Simpson | 0.91±0.54 | 0.91±0.12 | 0.63 |
| Observed OTUs | 884.7±143.08 | 852.5±134.11 | 0.61 |

Data are expressed as mean with standard error, n=10. CON=control group; HS=heat stress group.
