# Supplementary material for: Heat Stress Alters the Intestinal Microbiota and Metabolomic Profiles in Mice
Source: Front Microbiol. 2021 Aug 20;12:706772. doi: 10.3389/fmicb.2021.706772 (PMC8430895; doi:10.3389/fmicb.2021.706772)
Supplement: Supplementary Table 3 — Significantly different metabolites in cecal contents between CON and HS groups. [file Table_3.docx]

Table S3. Significantly different metabolites in cecal contents between CON and HS groups.

| Items | Groups | | *P*-value |
| --- | --- | --- | --- |
|  | CON | HS |  |
| Xanthine | 0.000928±0.0014 | 0.003478±0.0026 | 0.0132 |
| Stearic acid | 1.15861±0.3855 | 0.656761±0.2798 | 0.0037 |
| Shikimic acid | 0.002525±0.002 | 0.004388±0.0014 | 0.0263 |
| Salicin | 0.006356±0.0085 | 0.016342±0.0078 | 0.0137 |
| Purine riboside | 0.004158±0.0054 | 0.013796±0.0065 | 0.002 |
| Pipecolinic acid | 0.027554±0.0067 | 0.019296±0.0098 | 0.0413 |
| Palmitic acid | 1.454198±0.3516 | 1.101828±0.2725 | 0.0221 |
| Oleic acid | 0.22603±0.0562 | 0.161833±0.0488 | 0.0138 |
| Myristic acid | 0.031012±0.0069 | 0.021183±0.0119 | 0.0363 |
| Mannose | 0.040097±0.0532 | 1.143E-7±13E-9 | 0.0285 |
| Diglycerol | 0.00254±0.0025 | 0.005513±0.0027 | 0.0214 |
| Carbazole | 0.629387±0.1063 | 0.435771±0.1817 | 0.0094 |
| Behenic acid | 0.073913±0.0331 | 0.033359±0.0224 | 0.0049 |
| 4-Hydroxyphenylacetic acid | 0.009367±0.0044 | 0.004153±0.0052 | 0.0271 |
| 3-Aminopropionitrile | 0.012955±0.0009 | 0.007546±0.0066 | 0.0197 |
| 3,5-Dihydroxyphenylglycine | 0.00357±0.0075 | 0.011523±0.0084 | 0.0386 |
| 2-Deoxy-D-glucose | 0.012229±0.0111 | 0.025518±0.0129 | 0.0234 |

Data are expressed as mean with standard error. CON=control group; HS=heat stress group.
